# Knowledge of and attitudes towards hepatitis B and its transmission from mother to child among pregnant women in Guangdong Province, China

**DOI:** 10.1371/journal.pone.0178671

**Published:** 2017-06-02

**Authors:** Zhenyan Han, Yuzhu Yin, Yuan Zhang, Stephan Ehrhardt, Chloe L. Thio, Kenrad E. Nelson, Xiaoyi Bai, Hongying Hou

**Affiliations:** 1 Department of Obstetrics and Gynecology, the Third Affiliated Hospital of Sun Yat-Sen University, Guangzhou, Guangdong Province, China; 2 Department of Epidemiology, Johns Hopkins Bloomberg School of Public Health, Baltimore, United States of America; 3 Department of Medicine, Division of Infectious Diseases, Johns Hopkins University, Baltimore, United States of America; TNO, NETHERLANDS

## Abstract

**Background:**

Hepatitis B virus (HBV) infection remains a serious public health problem worldwide. Mother-to-child transmission (MTCT) of HBV is the major mode of transmission in HBV-endemic areas, including China, where little is known about pregnant women’s knowledge of and attitudes towards HBV infection and MTCT.

**Methods:**

A cross-sectional survey, conducted in pregnant women in Guangdong Province, China, measured HBV knowledge and attitudes using a questionnaire, at one tertiary and two rural hospitals.

**Results:**

The total response rate was 94.5% (737/780). Of the 11 knowledge questions, the mean score was 6.73 ± 3.04 (mean ± SD). Most pertinent to preventing MTCT, 53.3% of the respondents did not know that HBV can be transmitted through unprotected sexual intercourse and nearly 20% did not know that HBV can be transmitted from mother to infant. The results of the four attitude questions was better with 83% and 85% being willing to be screened for HBV and let their baby receive HBV vaccine and HBIg, respectively. However, only 16.5% of respondents agreed that they would be willing to take drugs that are known not to harm the fetus to prevent MTCT of HBV. In multivariable analysis, higher education level was associated with better knowledge and attitude scores.

**Conclusions:**

Knowledge about HBV among pregnant women was poor and needs to be improved to prevent MTCT of HBV. Health education needs to be directed towards pregnant mothers, particularly less educated mothers, in high HBV endemicity settings.

## Introduction

Hepatitis B virus (HBV) infection remains a serious global public health problem. Globally, there are an estimated 240 million people chronically infected with HBV, with more than 686,000 deaths annually due to complications of hepatitis B, including cirrhosis and hepatocellular carcinoma [1]. Hepatitis B prevalence is highest in sub-Saharan and East Asia, where 5–10% of population is chronically infected [1, 2]. In China, a 2006 national survey found a prevalence of hepatitis B surface antigen (HBsAg) to be 7.6% among women of childbearing age [3], and a recent study of 15 million couples in rural China demonstrated a seroprevalence of 5.2% in women 20–49 years old [4].

Mother-to-child-transmission (MTCT) is the major mode of HBV transmission worldwide, which is a problematic since around 90% of infected infants progress to chronic hepatitis B. This risk is much higher than from horizontal transmission where the rate of chronicity is 30–50% when infected before 6 years of age and <5% when infected in adulthood [1, 5, 6]. Despite improved childhood HBV vaccination worldwide, MTCT still accounts for about 50% of new HBV infections in high endemic countries and one-third in low endemic countries [7, 8, 9, 10]. Therefore, preventing MTCT is crucial for decreasing HBV prevalence. Prevention requires HBV-infected mothers to be aware of their disease status and to understand the consequences of HBV transmission to their child. Although, many studies have demonstrated that insufficient knowledge of HBV infection in the general public [11, 12, 13, 14, 15, 16] and among health care workers [17, 18, 19] is associated with high prevalence of hepatitis B, only a few studies have assessed knowledge of hepatitis B and MTCT among pregnant women [20, 21, 22]. These studies also indicated similar gaps of knowledge; however, they did not assess the attitudes of the mothers towards methods of preventing MTCT of hepatitis B. Their attitudes could affect their willingness for prenatal screening and to follow the current WHO immunoprophylaxis guidelines, which include birth dose vaccine, hepatitis B immunoglobulin for their infants, and completing HBV vaccine series prior to 1 year of age [23]. Therefore, this study addresses that gap by assessing attitudes towards screening and towards various interventions to prevent MTCT of HBV.

Guangdong Province, located in southern China, is a highly populated province, with 104 million people in 2010. The prevalence of HBsAg in general Guangdong population was 11.1% in 2006 [24] and 8.76% in 2015 [25], making this province a hotspot for HBV in China. Thus, this region allowed assessment of knowledge and attitudes towards HBV in a large number of pregnant women who had prenatal care in the outpatient departments of one of the three large hospitals–one tertiary care and two rural.

## Materials and methods

### Study design

The study was cross-sectional in design and recruited pregnant women from one of the following hospitals in Guangdong Province between May and October 2014: 1) the Third Affiliated Hospital of Sun Yat-Sen University (SYSU), which is a tertiary care hospital in Guangzhou City, 2) Panyu Maternal and Child Care Service Center (Panyu), and 3) Foshan Maternal and Child Hospital (Foshan). Panyu and Foshan are located in more rural areas of Guangdong Province. Women were invited to participate if they were pregnant, older than 18 years, and attending their first prenatal visit. Written informed consent in Chinese was obtained from the participants and the study was approved by the Institutional Review Boards at the Third Affiliated Hospital of Sun Yat-Sen University, China and the Johns Hopkins Bloomberg School of Public Health, Maryland, United States of America. Participants answered the questionnaire prior to receiving education about HBV infection.

At SYSU, the questionnaire was self-administered. At Panyu and Foshan, the questionnaire was administered via face-to-face interviews. This method was chosen because many women in these rural areas were not well educated so may have had difficulty finishing the questionnaire without assistance. The interviews were conducted by one of four trained staff that included one doctor, one nurse and two postgraduate students from the Obstetrics Department of SYSU. This study was elaborated according to STROBE guidelines (S3 File).

### Questionnaire

The questionnaire was jointly developed by the study team in English and then translated into Mandarin and back (S1 and S2 Files). Content and language were intensely discussed among the study team and pre-testing in pregnant Chinese women indicated that the questions were easy to understand. The questionnaire consisted of 21 items, divided into three parts: demographic information, knowledge of HBV, and attitudes about HBV. The demographic information included age, self-reported HBV infection status, number of children and education level. The knowledge section tested three aspects: 1) general knowledge of HBV (4 items: Q1, Q7, Q8, Q9); 2) modes of transmission (5 items: Q2, Q3, Q4, Q5, Q6); and 3) knowledge of vaccine and MTCT of HBV (2 items: Q10, Q11). The attitude section consisted of 6 items (Q12, Q13, Q14, Q15, Q16, Q17) and was mainly about prevention of MTCT of HBV and follow-up after birth. For each item there were three response options: ‘yes’, ‘no’, and ‘don’t know’. The English translation of the questionnaire is in Appendix 1.

### Data analysis

To avoid data entry errors, all data were entered into EpiData 3.1 separately by two postgraduate students and if there were inconsistencies between the two entries, then the correct entry was verified from the questionnaire. Each correct answer was given one point, and each incorrect answer or ‘don’t know’ was given zero points. Missing data were counted as incorrect. The overall knowledge score was the sum of the scores of 11 knowledge items. The attitude score was the sum of the six attitude items.

Descriptive analysis was used for baseline demographic information. Bivariate analysis (χ2 test and ANOVA) was used to test for differences across hospitals and participants. The association between knowledge or attitude scores and demographic information was assessed by linear regression and Spearman correlation was used to analyze the relationship between knowledge and attitude scores. *P* values <0.05 were considered significant. Data were analyzed using SPSS version 17 (SPSS Inc, USA).

## Results

### Characteristics of study participants

Of the 780 pregnant women invited to participate, 737 (94.5%) agreed and answered the questionnaire. The proportion who agreed to participate was high at all the sites: 92.4% (462/500) at SYSU, 98.1% (152/155) at Panyu and 98.4% (123/125) at Foshan. At SYSU, 12.6% (58/462) of questionnaires had missing data while there were no missing data from the other two hospitals.

Most of the participants were 26–35 years old (64.0%) and pregnant with their first baby (67.9%) (Table 1). Overall, 50.9% of the respondents attained an education level of college or above, and this proportion was highest at SYSU. Of the respondents, 10.0% reported having chronic hepatitis B, a proportion that was similar across the three hospitals.

**Table 1:** Characteristics of the study population.

Parameters	Total n (%)	n (%)			P
		SYSU	Panyu	Foshan	
**Age**					
18–25	237 (32.2)	133 (28.9)	57 (37.5)	47 (38.2)	<0.001
26–35	471 (64.0)	321 (69.6)	81 (53.3)	69 (56.1)	
>35	28 (3.8)	7 (1.5)	14 (9.2)	7 (5.7)	
Missing	1 (0.1)	1 (0.2)	0 (0.0)	0 (0.0)	
Valid Total	736 (99.9)	461 (99.8)	152 (100.0)	123 (100.0)	
**Prior live births**					
0	500 (67.9)	342 (74.2)	87 (57.2)	71 (57.7)	<0.001
≥1	236 (32.0)	119 (25.8)	65 (42.8)	52 (42.3)	
Missing	1 (0.1)	1 (0.2)	0 (0.0)	0 (0.0)	
Valid Total	736 (99.9)	461 (99.8)	152 (100.0)	123 (100.0)	
**Education level**					
Junior high or below	186 (25.2)	84 (18.2)	57 (37.5)	45 (36.6)	<0.001
Senior high	176 (23.9)	98 (21.2)	43 (28.3)	35 (28.5)	
College or above	375 (50.9)	280(60.6)	52 (34.2)	43 (35.0)	
Missing	0 (0.0)	0 (0.0)	0 (0.0)	0 (0.0)	
Valid Total	737 (100.0)	462 (100.0)	152 (100.0)	123 (100.0)	
**Self-reported chronic hepatitis B**					
Chronic hepatitis B	73 (10.0)	50 (10.9)	14 (9.2)	9 (7.3)	0.08
No chronic hepatitis B	621 (84.6)	377 (82.1)	132 (86.8)	112 (91.1)	
Unknown	40 (5.4)	32 (7.0)	6 (4.0)	2 (1.6)	
Missing	3 (0.4)	3 (0.6)	0 (0.0)	0 (0.0)	
Valid Total	734 (99.6)	459 (99.4)	152 (100.0)	123 (100.0)	

### Knowledge about HBV

The mean knowledge score was 6.73 ± 3.04 (mean ± SD) and the median was 7.0 (interquartile range (IQR) 5–9). Only 21.0% of participants were able to answer all the general knowledge questions correctly (Q1, Q7, Q8, Q9), including 43.3% who knew that infection with HBV could be asymptomatic (Q9)(Table 2). Regarding the various modes of transmission, only 21.6% answered all five questions (Q2, Q3, Q4, Q5, Q6) correctly. Moreover, pertinent to preventing MTCT of HBV, 53.3% of the respondents did not know that HBV may be transmitted through unprotected sexual intercourse and nearly 20% did not know that HBV could be transmitted from mother to infant. Furthermore, 60.2% did not know that HBV could be co-transmitted with HIV. The majority of participants (74.7%) knew that a hepatitis B vaccine was available in China. Only 58.8% were aware that MTCT of HBV could eventually lead to severe liver complications in either in childhood or as adults (Table 2).

**Table 2:** Responses to HBV knowledge questions, stratified by hospital.

items	Missing	Total correct answers, n (%)	Correct answers, n (%)			P
			SYSU	Panyu	Foshan	
**Q1**: Hepatitis B is caused by a virus	7 (0.9)	413 (56.6)	243 (53.4)	104 (68.4)	66 (53.7)	0.004
**Q2**: Hepatitis B can be transmitted through blood transfusion	2 (0.3)	548 (74.6)	331 (72.0)	127 (83.6)	90 (73.2)	0.016
**Q3:** Hepatitis B can be transmitted through unprotected sexual intercourse	4 (0.5)	342 (46.7)	224 (48.9)	68 (44.7)	50 (40.7)	0.23
**Q4:** Hepatitis B can be transmitted from mother to fetus	11 (1.5)	585 (80.6)	342 (75.8)	139 (91.4)	104 (84.6)	<0.001
**Q5:** Hepatitis B can be transmitted through use of unsafe needles or sharps	6 (0.8)	520 (71.1)	303 (66.4)	122 (80.3)	95 (77.2)	0.001
**Q6:** An individual can be infected by both Hepatitis B and HIV	9 (1.2)	290 (39.8)	196 (43.3)	53 (34.9)	41 (33.3)	0.051
**Q7:** Hepatitis B infection can lead to liver cancer	4 (0.5)	421 (57.4)	231 (50.4)	108 (71.1)	82 (66.7)	<0.001
**Q8:** Hepatitis B infection can lead to cirrhosis (scarred liver)	6 (0.8)	430 (58.8)	239 (52.4)	105 (69.1)	86 (69.9)	<0.001
**Q9:** A person can be infected with hepatitis B and not have any symptoms of the disease	9 (1.2)	315 (43.3)	175 (38.6)	89 (58.6)	51 (41.5	<0.001
**Q10:** There is a vaccine for hepatitis B	13 (1.8)	541 (74.7)	294 (65.5)	143 (94.1)	104 (84.6)	<0.001
**Q11:** Babies that are infected perinatally (at or around the time of delivery) are at high risk for eventual complications of liver fibrosis, cirrhosis or liver cancer	21 (2.8)	421 (58.8)	230 (52.2)	117 (77.0)	74 (60.2)	<0.001

In multivariate analysis, characteristics independently associated with higher knowledge scores included college or higher level of education, maternal age >35 years, and having self-reported chronic hepatitis B (Table 3). The number of prior live births was not associated with the knowledge score.

**Table 3:** Multivariable analysis of factors associated with knowledge scores.

Variable	Coef	95% CI	P
**Age (years) (ref: 18–25)**			
26–35	0.49	0.001–0.98	0.05
>35	1.36	0.16–2.55	0.03
**Prior live births (ref: 0)**			
≥1	0.18	-0.32–0.68	0.48
**Education (ref: junior high or below)**			
Senior high	0.48	-0.14–1.08	0.13
College or above	1.79	1.25–2.34	<0.001
**Self-reported chronic hepatitis B (ref: yes)**			
No	0.041	-0.75–0.67	0.91
Don’t know	-1.92	-3.05–0.78	0.001

### Attitudes towards HBV

A total of 68.5% of participants answered “yes” to all four questions regarding the willingness to be screened for HBV and to agree to the components of the current WHO recommendations to prevent MTCT of HBV (Q12, Q13, Q14, Q16). Of the respondents, 83.3% and 89.8% were willing to be screened for HBV during antenatal care and let their baby receive HBV vaccine, respectively (Table 4). Moreover, if they were diagnosed with HBV, 85.0% of them would let their baby receive HBIg and agree to HBV testing during the baby’s first year. However, only 49.0% of pregnant women were willing to allow blood draws from their child in the context of a clinical trial, and this proportion was lowest at SYSU hospital and highest at Panyu hospital. When asked about taking drugs in pregnancy to prevent MTCT of HBV, only 16.5% of respondents agreed that they would be willing to take drugs that are known not to harm the fetus.

**Table 4:** Comparison of positive attitudes (Yes response) towards HBV, stratified by hospital.

items	Missing	Total Yes response n (%)	Yes response, n (%)			P
			SYSU	Panyu	Foshan	
**Q12:** Are you willing to be screened for hepatitis B during an antenatal care visit (blood test)?	6 (0.8)	609 (83.3)	378 (82.9)	128 (84.2)	103 (83.7)	0.92
**Q13:** Are you willing to let your baby receive HBV vaccine?	1 (0.1)	661 (89.8)	397 (86.1)	148 (97.4)	116 (94.3)	<0.001
**Q14:** If you got HBV infection, are you willing to let your baby receive anti-HBV antibodies?	15 (2.0)	614 (85.0)	371 (83.0)	146 (96.1)	97 (78.9)	<0.001
**Q15:** If you got HBV infection, are you willing to take drugs that are known not to harm the developing baby in pregnancy to prevent transmitting HBV to your baby?	3 (0.4)	121 (16.5)	74 (16.1)	33 (21.7)	14 (11.4)	0.07
**Q16:** Are you willing to take your baby back to the clinic to test his/her HBV status a few times during the 1st year after birth?	3 (0.4)	624 (85.0)	376 (81.9)	144 (94.7)	104 (84.6)	0.001
**Q17:** If you got HBV infection, are you willing to let us draw blood from your child in the context of a clinical trial? (about 2 ml per visit; 5 visits)	3 (0.4)	360 (49.0)	176 (38.3)	121 (79.6)	63 (51.2)	<0.001

In multivariable analysis, a higher education level was the only factor independently associated with higher attitude scores (Table 5). Attitudes scores were weakly correlated with knowledge scores (correlation 0.352, *P*<0.001).

**Table 5:** Multivariable analysis of factors associated with attitude scores.

Variables	Coef	95% CI	P
**Age (years) (ref: 18–25)**			
26–35	-0.11	-0.35–0.13	0.36
>35	0.20	-0.38–0.78	0.50
**Prior live births (ref: 0)**			
≥1	0.075	-0.17–0.32	0.54
**Education (ref: junior high or below)**			
Senior high	0.33	0.035–0.62	0.028
College or above	0.45	0.19–0.72	0.001
**Self-reported chronic hepatitis B (ref: yes)**			
No	-0.22	-0.57–0.12	0.20
Don’t know	-0.33	-0.88–0.228	0.24

## Discussion

This study supports that insufficient knowledge about HBV is a potential barrier to eliminating MTCT since a minority of women correctly answered all the general HBV knowledge questions or all the questions about HBV transmission. This lack of knowledge may also influence the attitudes of the mother towards interventions that could reduce the risk of transmission to their infants. Our findings are not limited to this region of China since insufficient knowledge in various aspects of HBV was similarly found in other studies of pregnant women in Hong Kong and other high endemic areas [20, 21, 22].

Important for MTCT, about half of the women did not know that chronic infection may be asymptomatic, and did not recognize that HBV may be transmitted through unprotected sexual intercourse. Notably, almost 20% did not know that they could transmit HBV to their infant. These data are consistent with similar studies among pregnant women from other high endemic regions [20, 21, 22]. Thus, basic education about HBV is needed in pregnant women since lack of knowledge is a barrier towards making decisions about protecting their infants from HBV.

Interestingly, participants in this study were less knowledgeable about HBV when compared to Chinese and Vietnamese immigrants living in areas of the world with low HBV endemicity [13, 26, 27]. This knowledge deficit amongst those living in China may be related to not including HBV as a sexually transmitted disease in public health promotion and educational materials in China. Furthermore, despite the fact that HBV vaccine was fully integrated into the expanded routine immunization program freely available to all infants from 2002, only 74.7% of our survey participants were aware that hepatitis B vaccine was routinely available in China. This rate is much lower when compared with 95% of immigrant Asian Americans who understand that HBV is preventable by vaccination [13, 27]. Taken together, these data indicate that increased efforts to disseminate information about hepatitis B are needed and especially to young women of child-bearing age if the goal is to eliminate MTCT of HBV in China. Our multivariable analysis support that this information needs to be in simplified language since higher HBV knowledge scores were associated with higher education levels. People with more education likely have greater access to information from various sources including mass media, health websites, educational pamphlets, and healthcare professionals, and they are more likely to understand and interpret health information more readily.

The lack of knowledge is one explanation why only 16.5% of participants expressed willingness to take antiviral agents that are safe in pregnancy to prevent MTCT of HBV. This result is consistent with another survey conducted in China in 2011 that found 11.7% of obstetric and gynecology staff thought antiviral therapy was important during pregnancy [17]. These data are also consistent with a recent prospective study at SYSU of telbivudine treatment during pregnancy to prevent HBV MTCT where only 29.9% of pregnant women with high HBV DNA levels voluntarily accepted antiviral therapy [28]. In order to increase the willingness of women to take antivirals during pregnancy, further work is needed to educate women about both the long-term consequences of HBV infection in an infant and about prevention of MTCT of HBV.

Our study had several limitations. The three hospitals are located in a highly endemic and relatively developed area; thus, the results may not be applicable to other areas of the world. Also, administration mode for the questionnaire was not uniform at all sites, so this may have increased heterogeneity of data and decreased the comparability of the results between rural and urban areas. Lastly, the self-reported HBV infection status data could not be validated.

## Conclusion

Our survey found that pregnant women had insufficient knowledge regarding HBV infection. Despite most respondents being aware of the importance of antenatal screening, neonatal vaccination and postnatal follow-up of HBV, very few were willing to receive antiviral therapy to prevent MTCT of HBV. This deficiency in knowledge and attitudes was most prominent in less educated women. Additional efforts to enhance HBV public health education programs in understandable language are needed to achieve the goal of eliminating MTCT of HBV. Future studies could be aimed towards determining the impact of such education programs.

## Supporting information

S1 File KAP_Pregnant women- English version. (PDF)

S2 File KAP_Pregnant women- Chinese version. (PDF)

S3 File STROBE_checklist_v4_combined_PlosMedicine. (DOCX)
