# Supplementary material for: Knowledge of and attitudes towards hepatitis B and its transmission from mother to child among pregnant women in Guangdong Province, China
Source: PLoS One. 2017 Jun 2;12(6):e0178671. doi: 10.1371/journal.pone.0178671 (PMC5456270; doi:10.1371/journal.pone.0178671)
Supplement: S1 File — (PDF) [file pone.0178671.s001.pdf]

Questionnaire about mother to child transmission of hepatitis B

Consent for Survey of Pregnant women

PI Name: Hongying Hou, Chaoshuang Lin, Stephan Ehrhardt, Chloe L Thio, Kenrad Nelson

Study Title: Knowledge about mother to child transmission of hepatitis B survey.

IRB No.: [2013]2-120/IRB00005636

PI Version/Date: 2014-1-6

Hello. You have been invited to participate in a survey on the knowledge and attitudes with respect to hepatitis B, its prevention, and its treatment among pregnant women. This study is being done by Sun Yat-sen University and the Johns Hopkins University. Your answer will be important for us to understand what pregnant women in China think about HBV.

This is an anonymous study, and your participation is completely voluntary. We will not disclose your answers to anyone else outside our research team. We will do our best to keep your information safe by not writing down your name and using a special code on the questionnaire. We will also keep all questionnaire locked in a filing cabinet at infectious department. If we share your information with other researchers, they will use the same protections.

It will take about 10 minutes to answer the questions. It is OK to choose “don’t know” if you don’t know the answer. If you have any questions during the survey, you may ask the study coordinator to explain/clarify. You do not have to answer all the questions and you may stop at any time.

If you have any questions after the survey, you may contact Weiqiang Gan (86-20-13560195664, ganweiqiang@aliyun.com) or Siying Liao (86-20-18922103021, seeingseeing@163.com) about your questions or problems with this work.

Please note that by answering the questions and turning in the questionnaire, you have given your consent to participate in the study.

Questionnaire about mother to child transmission of hepatitis B

Demographics and Basic information

Age: 1=18-25, 2= 26-35, 3= 36-45, 4= 46-55, 5= 56-65

Do you have HBV infection? 1= Yes, 2= No, 3= Don’t know

How many children do you have (do not include the current pregnancy)? 1= 0, 2= 1, 3= more than 1

Education: 1= never go to school, 2= primary school, 3= junior school, 4= senior school, 5= collage, 6= master or above

Knowledge

	Yes	No	Don’t know
Hepatitis B is caused by a virus			
Hepatitis B can be transmitted through blood transfusion			
Hepatitis B can be transmitted through unprotected sexual intercourse			
Hepatitis B can be transmitted from mother to fetus			
Hepatitis B can be transmitted through use of unsafe needles or sharps			
An individual can be infected by both Hepatitis B and HIV			
Hepatitis B infection can lead to liver cancer			
Hepatitis B infection can lead to cirrhosis (scarred liver)			
A person can be infected with hepatitis B and not have any symptoms of the disease			
There is a vaccine for hepatitis B			
Babies that are infected perinatally (at or around the time of delivery) are at high risk for eventual complications of liver fibrosis, cirrhosis or liver cancer			

Questionnaire about mother to child transmission of hepatitis B

Attitudes

	Yes	No	Don't know
Are you willing to be screened for hepatitis B during an antenatal care visit (blood test)?			
Are you willing to let your baby receive HBV vaccine?			
If you got HBV infection, are you willing to let your baby receive anti-HBV antibodies?			
If you got HBV infection, are you willing to take drugs that are known not to harm the developing baby in pregnancy to prevent transmitting HBV to your baby?			
Are you willing to take your baby back to the clinic to test his/her HBV status a few times during the 1st year after birth?			
If you got HBV infection, are you willing to let us draw blood from your child in the context of a clinical trial? (about 2 ml per visit; 5 visits)			
