# Supplementary material for: Knowledge of and attitudes towards hepatitis B and its transmission from mother to child among pregnant women in Guangdong Province, China
Source: PLoS One. 2017 Jun 2;12(6):e0178671. doi: 10.1371/journal.pone.0178671 (PMC5456270; doi:10.1371/journal.pone.0178671)
Supplement: S2 File — (PDF) [file pone.0178671.s002.pdf]

乙肝病毒母婴传播相关知识的调查问卷

孕妇知情同意书

项目负责人姓名：侯红瑛，林潮双，Stephan Ehrhardt, Chloe L Thio, Kenrad Nelson

项目名称：乙肝病毒母婴垂直传播知识调查问卷

IRB No.：[2013]2-120/IRB00005636

版本/日期：2014-1-6

您好！

您已被邀请参与关于孕妇慢性乙型肝炎的预防和治疗的知识和态度调查。该项研究由中山大学和约翰斯·霍普金斯大学共同开展。您的答案对我们了解中国孕妇的 HBV 认知程度非常重要。

这是一项匿名调查，您的参与完全是自愿的。我们不会将您的答案向我们的研究团队以外的任何人泄露。我们将通过匿名和对问卷使用特殊编码的形式保证您的资料安全。同时我们将把所有问卷统一锁放在感染科的文档柜中。研究人员在使用您的信息时都将遵循以上的保护措施。因此，您参与本研究不会发生任何风险。

您将需要约 10 分钟时间来回答所有问题。如果您不知道答案，您可以选择“不知道”。如果您对任何问题有不明白的地方，可以咨询研究协调员，他们将向您解答。因为本调查完全是自愿的，您不是必须回答所有问题，您随时有权拒绝参与及退出研究。

调查之后如果您对有任何问题，您可以联系淦伟强 (86-20-13560195664, ganweiqiang@aliyun.com) 或廖思莹 (86-20-18922103021, seeingseeing@163.com)。

请您注意，当您回答问题并交回问卷时，就证明您已经同意参加这项研究了。

乙肝病毒母婴传播相关知识的调查问卷

基本资料

**年龄:** 1 = 18-25，2= 26-35，3= 36-45，4= 46-55，5= 56-65
**您是否有乙肝病毒感染?** 1= 有，2= 没有，3= 不知道
**您有几个孩子** (不包括现在怀孕的这胎)? 1=0 个，2=1 个，3=1 个以上
**教育程度：**1=文盲，2=小学，3=初中，4=高中，5=大专或本科，6=硕士或以上

知识

	是	不是	不知道
乙型肝炎是由病毒引起的			
乙型肝炎可以通过血液传播			
乙型肝炎可以通过无保护性行为传播			
乙型肝炎可以通过母亲传播给胎儿			
乙型肝炎可以通过不安全的针头或尖锐物传播			
一个人可以同时感染乙型肝炎和艾滋病			
乙型肝炎可以导致肝癌			
乙型肝炎可以导致肝硬化			
病人感染乙型肝炎时可以没有任何症状			
目前是有乙肝疫苗的			
宝宝从母亲那里感染乙肝（在分娩时或者接近分娩时），今后并发肝纤维化，肝硬化和肝癌的风险更高			

Attitudes 态度

	愿意	不愿意	不知道
产前检查时您是否愿意接受乙型肝炎相关的血液检查?			
您是否愿意让您的宝宝注射乙肝疫苗？			
假设您感染了乙肝，您是否愿意让您的宝宝注射抗乙肝抗体？			
假设您感染了乙肝，为了防止将乙肝传染给宝宝，您愿意接受那些对宝宝发育无害的药物治疗？			
您是否愿意带宝宝在生后第一年内回医院检查宝宝的乙肝病染情况？			
假设您感染了乙肝，您是否愿意让我们在临床研究过程中为您的宝宝抽血检查？（大概抽 5 次血，每次 2 毫升）			
