# Supplementary material for: Knowledge of and attitudes towards hepatitis B and its transmission from mother to child among pregnant women in Guangdong Province, China
Source: PLoS One. 2017 Jun 2;12(6):e0178671. doi: 10.1371/journal.pone.0178671 (PMC5456270; doi:10.1371/journal.pone.0178671)
Supplement: S3 File — (DOCX) [file pone.0178671.s003.docx]

STROBE Statement—checklist of items that should be included in reports of observational studies

|  | Item No. | Recommendation | | Page  No. | Relevant text from manuscript |
| --- | --- | --- | --- | --- | --- |
| **Title and abstract** | 1 | (*a*) Indicate the study’s design with a commonly used term in the title or the abstract | | 2 | A cross-sectional survey, conducted in pregnant women in Guangdong Province, China, measured HBV knowledge and attitudes using a questionnaire, at one tertiary and two rural hospitals. |
|  |  | (*b*) Provide in the abstract an informative and balanced summary of what was done and what was found | | 3 | Knowledge about HBV among pregnant women was poor and needs to be improved to prevent MTCT of HBV. Health education needs to be directed towards pregnant mothers, particularly less educated mothers, in high HBV endemicity settings. |
| Introduction | | | | |  |
| Background/rationale | 2 | Explain the scientific background and rationale for the investigation being reported | | 3-4 | Mother-to-child-transmission (MTCT) is the major mode of HBV transmission worldwide, leading to chronic hepatitis B in around 90% of infected infants. Only a few studies have assessed knowledge of hepatitis B and MTCT among pregnant women. These studies indicated similar gaps of knowledge; however, they did not assess the attitudes of the mothers towards methods of preventing MTCT of hepatitis B. Their attitudes could affect their willingness for prenatal screening and to follow the current WHO immunoprophylaxis guidelines, which include birth dose vaccine, hepatitis B immunoglobulin for their infants, and completing HBV vaccine series prior to 1 year of age. |
| Objectives | 3 | State specific objectives, including any prespecified hypotheses | | 4 | As a first step towards increasing efforts to prevent MTCT of HBV, this study measured HBV knowledge of HBV among pregnant women and addressed gap by assessing attitudes towards screening and towards various interventions to prevent MTCT of HBV. |
| Methods | | | | |  |
| Study design | 4 | Present key elements of study design early in the paper | | 5-6 | The questionnaire was jointly developed by the study team in English and then translated into Mandarin and back. Content and language were intensely discussed among the study team and pre-testing in pregnant Chinese women indicated that the questions were easy to understand. The questionnaire consisted of 21 items, divided into three parts: demographic information, knowledge of HBV, and attitudes about HBV. |
| Setting | 5 | Describe the setting, locations, and relevant dates, including periods of recruitment, exposure, follow-up, and data collection | | 5-7 | The study was cross-sectional in design and recruited pregnant women from one of the following hospitals in Guangdong Province between May and October 2014: 1) the Third Affiliated Hospital of Sun Yat-Sen University (SYSU), which is a tertiary care hospital in Guangzhou City, 2) Panyu Maternal and Child Care Service Center (Panyu), and 3) Foshan Maternal and Child Hospital (Foshan). Panyu and Foshan are located in more rural areas of Guangdong Province. To avoid data entry errors, all data were entered into EpiData 3.1 using double-checking strategy by two postgraduate students and analyzed using SPSS version 17 (SPSS Inc, USA). |
| Participants | 6 | (*a*) *Cohort study*—Give the eligibility criteria, and the sources and methods of selection of participants. Describe methods of follow-up  *Case-control study*—Give the eligibility criteria, and the sources and methods of case ascertainment and control selection. Give the rationale for the choice of cases and controls  *Cross-sectional study*—Give the eligibility criteria, and the sources and methods of selection of participants | | 5 | Women were invited to participate if they were pregnant, older than 18 years, and attending their first prenatal visit. At SYSU, the questionnaire was self-administered. At Panyu and Foshan, the questionnaire was administered via face-to-face interviews. |
|  |  | (*b*) *Cohort study*—For matched studies, give matching criteria and number of exposed and unexposed  *Case-control study*—For matched studies, give matching criteria and the number of controls per case | | N/A |  |
| Variables | 7 | Clearly define all outcomes, exposures, predictors, potential confounders, and effect modifiers. Give diagnostic criteria, if applicable | | 6 | Each correct answer was given one point, and each incorrect answer or ‘don’t know’ was given zero points. Missing data were counted as incorrect. The overall knowledge score was the sum of the scores of 11 knowledge items. The attitude score was the sum of the six attitude items. |
| Data sources/ measurement | 8* | For each variable of interest, give sources of data and details of methods of assessment (measurement). Describe comparability of assessment methods if there is more than one group | | 6 | Each correct answer was given one point, and each incorrect answer or ‘don’t know’ was given zero points. Missing data were counted as incorrect. The overall knowledge score was the sum of the scores of 11 knowledge items. The attitude score was the sum of the six attitude items. |
| Bias | 9 | Describe any efforts to address potential sources of bias 5 | |  | Women were invited to participate if they were pregnant, older than 18 years, and attending their first prenatal visit. Participants answered the questionnaire prior to receiving education about HBV infection. The interviews were conducted by one of four trained staff that included one doctor, one nurse and two postgraduate students from the Obstetrics Department of SYSU. |
| Study size | 10 | Explain how the study size was arrived at | 4, 7 | | Guangdong Province, located in southern China, is a highly populated province, with 104 million people in 2010. The prevalence of HBsAg in general Guangdong population was 11.1% in 2006 [24] and 8.76% in 2015 [25], making this province a hotspot for HBV in China. In this study, of the 780 pregnant women invited to participate, 737 (94.5%) agreed and answered the questionnaire. |

Continued on next page

| Quantitative variables | 11 | Explain how quantitative variables were handled in the analyses. If applicable, describe which groupings were chosen and why | N/A |  |
| --- | --- | --- | --- | --- |
| Statistical methods | 12 | (*a*) Describe all statistical methods, including those used to control for confounding | 6-7 | Descriptive analysis was used for baseline demographic information. Bivariate analysis (χ2 test and ANOVA) was used to test for differences across hospitals and participants. The association between knowledge or attitude scores and demographic information was assessed by linear regression and Spearman correlation was used to analyze the relationship between knowledge and attitude scores. Data were analyzed using SPSS version 17 (SPSS Inc, USA). |
|  |  | (*b*) Describe any methods used to examine subgroups and interactions | 5-6 | The association between knowledge scores and demographic information was assessed by linear regression and Spearman correlation was used to analyze the relationship between knowledge and attitude scores. |
|  |  | (*c*) Explain how missing data were addressed | 5 | Each correct answer was given one point, and each incorrect answer or ‘don’t know’ was given zero points. Missing data were counted as incorrect. |
|  |  | (*d*) *Cohort study*—If applicable, explain how loss to follow-up was addressed  *Case-control study*—If applicable, explain how matching of cases and controls was addressed  *Cross-sectional study*—If applicable, describe analytical methods taking account of sampling strategy | N/A | T |
|  |  | (*e*) Describe any sensitivity analyses | 5 | P values <0.05 were considered significant. |
| Results | | | | |
| Participants | 13* | (a) Report numbers of individuals at each stage of study—eg numbers potentially eligible, examined for eligibility, confirmed eligible, included in the study, completing follow-up, and analysed | 7 | Of the 780 pregnant women invited to participate, 737 (94.5%) agreed and answered the questionnaire. The proportion who agreed to participate was high at all the sites: 92.4% (462/500) at SYSU, 98.1% (152/155) at Panyu and 98.4% (123/125) at Foshan. |
|  |  | (b) Give reasons for non-participation at each stage | N/A |  |
|  |  | (c) Consider use of a flow diagram | N/A |  |
| Descriptive data | 14* | (a) Give characteristics of study participants (eg demographic, clinical, social) and information on exposures and potential confounders | 7 | Most of the participants were 26-35 years old (64.0%) and pregnant with their first baby (67.9%) (Table 1). Overall, 50.9% of the respondents attained an education level of college or above, and this proportion was highest at SYSU. Of the respondents, 10.0% reported having chronic hepatitis B, a proportion that was similar across the three hospitals. |
|  |  | (b) Indicate number of participants with missing data for each variable of interest | 7 | At SYSU, 12.6 % (58/462) of questionnaires had missing data while there were no missing data from the other two hospitals. |
|  |  | (c) *Cohort study*—Summarise follow-up time (eg, average and total amount) | N/A |  |
| Outcome data | 15* | *Cohort study*—Report numbers of outcome events or summary measures over time | N/A |  |
|  |  | *Case-control study—*Report numbers in each exposure category, or summary measures of exposure | N/A |  |
|  |  | *Cross-sectional study—*Report numbers of outcome events or summary measures | 7 | Of 780 pregnant women who were invited, 737 (94.5%) agreed to participate. |
| Main results | 16 | (*a*) Give unadjusted estimates and, if applicable, confounder-adjusted estimates and their precision (eg, 95% confidence interval). Make clear which confounders were adjusted for and why they were included | 9-11 | Table 3 and table 5 |
|  |  | (*b*) Report category boundaries when continuous variables were categorized | 8 | mean ± SD |
|  |  | (*c*) If relevant, consider translating estimates of relative risk into absolute risk for a meaningful time period | N/A |  |

Continued on next page

| Other analyses | 17 | Report other analyses done—eg analyses of subgroups and interactions, and sensitivity analyses | N/A |  |
| --- | --- | --- | --- | --- |
| Discussion | | | | |
| Key results | 18 | Summarise key results with reference to study objectives | 11-13 | This study supports that insufficient knowledge about HBV is a potential barrier to eliminating MTCT since a minority of women correctly answered all the general HBV knowledge questions or all the questions about HBV transmission. Our findings are not limited to this region of China since insufficient knowledge of various aspects of HBV was similarly found in other studies of pregnant and fertile women in Hong Kong and other high endemic areas. Important for MTCT, the lack knowledge about that HBV chronic infection may be asymptomatic, transmitted through unprotected sexual intercourse and MTCT are consistent with similar studies among pregnant women from other high endemic regions. Our multivariable analysis support that this information needs to be in simplified language since higher HBV knowledge scores were associated with higher education levels. |
| Limitations | 19 | Discuss limitations of the study, taking into account sources of potential bias or imprecision. Discuss both direction and magnitude of any potential bias | 13-14 | The three hospitals are located in a highly endemic and relatively developed area; thus, the results may not be applicable to other areas of the world. Also, administration mode for the questionnaire was not uniform at all sites, so this may have increased heterogeneity of data and decreased the comparability of the results between rural and urban areas. Lastly, the self-reported HBV infection status data could not be validated. |
| Interpretation | 20 | Give a cautious overall interpretation of results considering objectives, limitations, multiplicity of analyses, results from similar studies, and other relevant evidence | 14 | Our survey found that pregnant women had insufficient knowledge regarding HBV infection. Despite most respondents being aware of the importance of antenatal screening, neonatal vaccination and postnatal follow-up of HBV, very few were willing to receive antiviral therapy to prevent MTCT of HBV. This deficiency in knowledge and attitudes was most prominent in less educated women. Additional efforts to enhance HBV public health education programs in understandable language are needed to achieve the goal of eliminating MTCT of HBV. |
| Generalisability | 21 | Discuss the generalisability (external validity) of the study results | N/A |  |
| Other information | |  | | |
| Funding | 22 | Give the source of funding and the role of the funders for the present study and, if applicable, for the original study on which the present article is based |  | CLT, KEN and SE were funded by the Johns Hopkins University Pilot grant. The funders had no role in study design, data collection and analysis, decision to publish, or preparation of the manuscript. |

*Give information separately for cases and controls in case-control studies and, if applicable, for exposed and unexposed groups in cohort and cross-sectional studies.

**Note:** An Explanation and Elaboration article discusses each checklist item and gives methodological background and published examples of transparent reporting. The STROBE checklist is best used in conjunction with this article (freely available on the Web sites of PLoS Medicine at http://www.plosmedicine.org/, Annals of Internal Medicine at http://www.annals.org/, and Epidemiology at http://www.epidem.com/). Information on the STROBE Initiative is available at www.strobe-statement.org.
